# Groove Pancreatitis: A Case Report and Review of a Hidden Type of Chronic Pancreatitis

**DOI:** 10.7759/cureus.27738

**Published:** 2022-08-06

**Authors:** Shweta A Kutty, Ravindran Chirukandath, Babu PJ, Nimisha C, Ancy T A

**Affiliations:** 1 General Surgery, Government Medical College and Hospital, Thrissur, IND; 2 Research, Government Medical College and Hospital, Thrissur, IND

**Keywords:** whipple’s pancreaticoduodenectomy, pancreatic cancer, pancreatic adenocarcinoma, surgical gastro, pancreatic surgery, hepato pancreato biliary surgery, general gastroenterology, duodenal obstruction, chronic pancreatitis, groove pancreatitis

## Abstract

Groove pancreatitis is a chronic type of segmental or focal pancreatitis seen to affect the groove, which is the region between the head of the pancreas, the duodenum, and the common bile duct. Despite its incidence remaining unknown, it accounts for 2.7% to 24.5% of pancreaticoduodenectomies performed for chronic pancreatitis. A diverse etiology has been implicated but the exact cause is yet to be identified. As it closely mimics pancreatic malignancy and remains mostly undiagnosed preoperatively, many patients often end up undergoing a pancreaticoduodenectomy. Awareness of this entity and early diagnosis will help us address this issue with more conservative measures than by resorting to a morbid procedure such as a pancreaticoduodenectomy.

We report a case of a 50-year-old male, a chronic alcoholic, with a two-year history of upper abdominal pain, postprandial vomiting, and weight loss. An abdominal contrast-enhanced computed tomography (CECT) scan was suggestive of either a pancreatic malignancy or a possibility of groove pancreatitis. However, postoperative histopathological examination confirmed the lesser known groove pancreatitis. Here, we review the clinical, radiological, and pathological characteristics of groove pancreatitis, as its diagnosis and management still pose a challenge.

## Introduction

Groove pancreatitis is a chronic type of segmental or focal pancreatitis affecting the groove region between the head of the pancreas, the duodenum, and the common bile duct. It was first reported by Becker in 1973 [1]. Despite its incidence remaining unknown, it accounts for 2.7% to 24.5% of pancreaticoduodenectomies performed for chronic pancreatitis. It mostly affects males between 40 and 50 years. The exact etiology is yet to be determined but associations include a history of long-term alcoholism, smoking, pancreatic duct obstruction, Brunner gland hyperplasia, and ectopic pancreatic tissue. It closely mimics pancreatic malignancy and remains mostly undiagnosed preoperatively. This is because it is difficult to differentiate between the two entities even on the basis of clinical, radiological, and laboratory parameters, thereby subjecting most patients to a morbid procedure - a pancreaticoduodenectomy [2-4]. Awareness of this entity and its early diagnosis can help manage this condition using conservative measures.

## Case presentation

A 50-year-old male with a history of consuming large amounts of alcohol and smoking for 30 years presented with chronic epigastric pain of two years duration. It was associated with the postprandial, non-bilious type of vomiting, weight loss, and loss of appetite over the recent year. Examination revealed epigastric tenderness and a lump of size 4x5 cm. His serum amylase and lipase were 80U/L and 101U/L, respectively.

**Figure 1:**
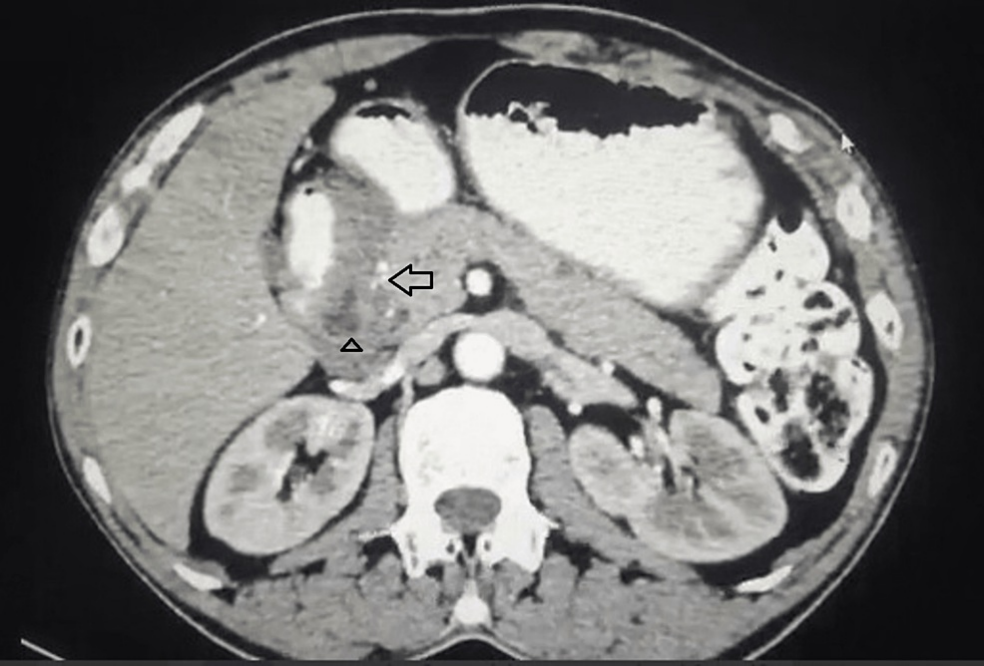
Contrast-enhanced CT scan showing a heterogeneously enhancing mass lesion involving the head of the pancreas (arrow) and the second part of the duodenum (D2) with cystic spaces (triangle)

A contrast-enhanced CT (computed tomography) scan of the abdomen and pelvis revealed an asymmetric, heterogeneously enhancing, hypodense lesion involving the pancreaticoduodenal groove causing thickening of the medial wall of the second part of the duodenum (D2) with luminal narrowing and few cystic areas within it, having a mass effect on the common bile duct (CBD). The carbohydrate antigen 19-9 (CA 19-9), carcinoembryonic antigen (CEA), and bilirubin levels were normal (Figure 1).

**Figure 2:**
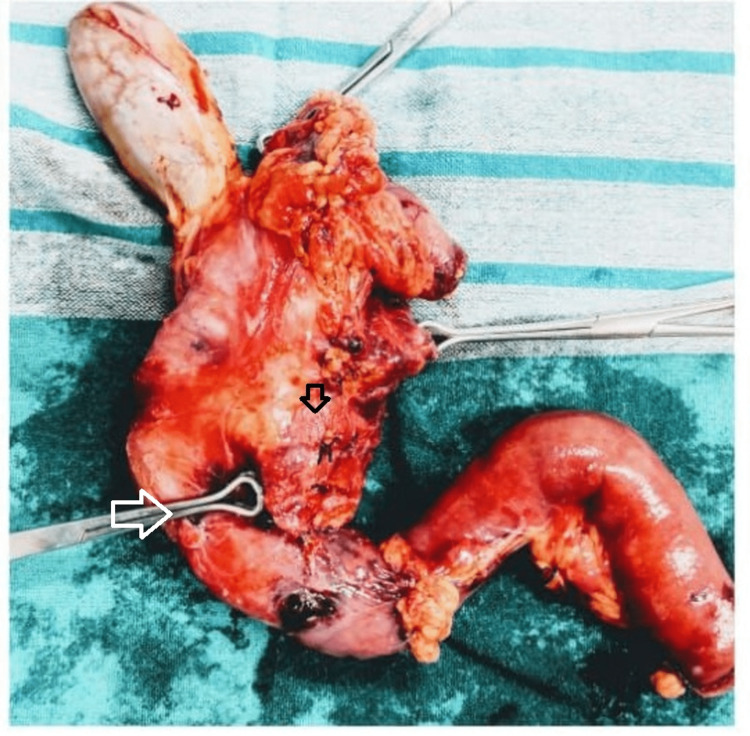
Resected Whipple's pancreaticoduodenectomy specimen showing a lesion arising from the pancreatic head (black arrow) and causing narrowing of D2 (white arrow)

An upper GI endoscopy revealed a bulge in the second part of the duodenum (D2) with luminal narrowing. Intraoperatively; a 4x4 cm hard mass originating from the head of the pancreas causing narrowing of D2 with suspicion of extension into the distal CBD was also noted. The lesion was operable and malignancy could not be ruled out on the table as an intraoperative frozen section was not done. Hence, a decision to perform Whipple’s pancreaticoduodenectomy was made. The repair consisted of a pancreaticojejunostomy, choledochojejunostomy, gastrojejunostomy, and finally a Roux-en-Y jejunojejunostomy (Figure 2).

**Figure 3:**
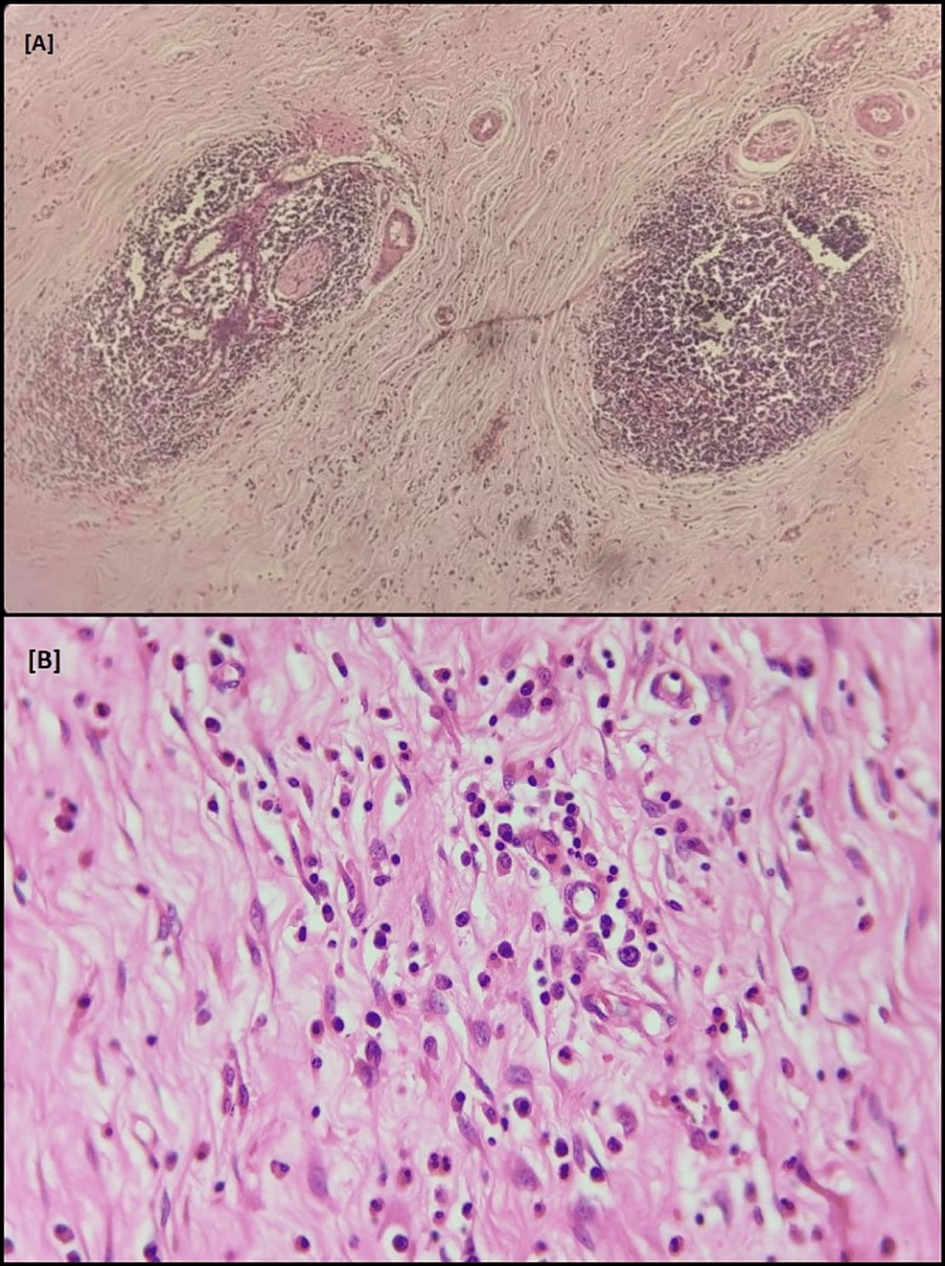
[A] Microscopy showing diffuse fibrosis seen as myofibroblastic infiltration; [B] A myofibroblastic infiltrate seen as spindle cells with occasional nuclear atypia

The histopathological report of the specimen revealed chronic inflammation and fibrosis with cystic areas involving the region between the pancreatic head and duodenum consistent with groove pancreatitis (Figure 3).

The postoperative period was uneventful, and the patient made a full recovery.

## Discussion

“Rinnenpankreatitis” was the first name attributed to this entity by Becker in 1973 [1]. Stolte et al. in 1982 used the term “Groove Pancreatitis” to describe chronic pancreatitis affecting the groove region [2]. Groove pancreatitis contributes to roughly 2.7% to 24.5% of chronic pancreatitis-associated pancreaticoduodenectomies performed.

Further classified by Becker and Mischke into two forms - a pure form and a segmental form. The pure form is confined only to the groove. The pancreatic parenchyma and the main pancreatic ducts are uninvolved. In the segmental form, the groove, as well as the head of the pancreas, are involved, causing stenosis of the terminal pancreatic duct with upstream dilatation [2-3].

The numerous names attributed to this condition include paraduodenal pancreatitis, cystic dystrophy of the heterotopic pancreas, paraduodenal wall cyst, pancreatic hamartoma of the duodenum, and myoadenomatosis.

Groove pancreatitis poses a diagnostic dilemma, as it clinically and radiologically mimics a pancreatic head, duodenal, or ampullary malignancy. The exact etiology is yet to be determined but associations include a history of long-term alcoholism and/or smoking, pancreatic duct obstruction, Brunner gland hyperplasia, and ectopic pancreatic tissue, all of which alter the anatomy and function at the minor papilla [3-5].

Groove pancreatitis can present either similar to acute pancreatitis or chronic pancreatitis and is commonly seen in males between 40 and 50 years of age [5-6].

Serological tests often show a normal to slight elevation of serum pancreatic enzymes and sometimes an occasional rise of serum hepatic enzymes [7]. Tumor markers, such as carcinoembryonic antigen (CEA) and carbohydrate antigen 19-9 (CA-19-9), are usually within the normal range [8].

Contrast-enhanced CT scan findings in groove pancreatitis include a hypodense lesion in the groove region, multiple cysts located within a thickened duodenal wall exhibiting post-contrast enhancement, or the involvement of the medial duodenal wall causing luminal obstruction, calcifications, pancreatic duct and common bile duct obstruction, and proximal dilatation [9].

On magnetic resonance imaging (MRI), it is identified as a sheet-like mass between the pancreatic head and the duodenum. Delayed enhancement on gadolinium-enhanced MRI images reflects the fibrous nature of the lesion. Cystic lesions of the groove and duodenal wall may also be seen on T2-weighted images.

Magnetic resonance cholangiopancreatography (MRCP) may reveal a dilated or normal main pancreatic duct (MPD) or a dilation at the ampulla of Vater. MRCP is sensitive to assess anomalies of the CBD, pancreatic duct (PD), and ampulla and may also reveal an increase in the distance between the duodenal lumen and distal duct [10].

Endoscopic ultrasound (EUS) represents one of the most sensitive methods for the detection of pancreaticobiliary pathologies. EUS can detect thickening or stenosis at the second part of the duodenum, intramural cysts, stenosis of CBD, masses arising in the groove region or pancreatic head, presence of calcifications or pseudocysts, and dilatation of the MPD. Additionally, EUS offers the advantage of a nonoperative tissue diagnosis - the guided fine needle aspiration cytology (FNAC), which can help differentiate pancreatic adenocarcinoma from chronic pancreatitis [11].

In the absence of severe symptoms, a prospective diagnosis may favor a conservative line of management which includes keeping the patient nil per oral, complete abstinence from smoking and alcohol consumption allowing pancreatic functions to recover along with adequate analgesia [12].

If these measures fail, endoscopic interventions are an alternative. They include endoscopic drainage of the minor papilla or pancreatic duct stenting, which are both less invasive than surgery [13-14].

However, in the presence of severe symptoms (similar to chronic pancreatitis - intractable pain, duodenal obstruction, jaundice), inability to distinguish from malignancy, or in the failure of all other treatment modalities, surgery is the only mainstay. The surgical treatment of choice is either Whipple’s procedure or pylorus-preserving pancreaticoduodenectomy [15].

## Conclusions

To distinguish groove pancreatitis clinically or radiologically from a primary pancreatic, duodenal or ampullary malignancy still remains a challenge thus making an early diagnosis difficult and often leading to a morbid surgery. Making a prospective diagnosis still poses a dilemma, as most cases of groove pancreatitis are diagnosed after a histopathological examination post-surgical excision. Newer studies suggest a more conservative or less invasive endoscopic approach for the condition. Surgical excision is needed only if all measures fail to provide adequate symptomatic relief or if there is a suspicion of malignancy. However, this is only possible if the diagnosis can be made prospectively rather than retrospectively.

## References

[1] Becker V (1973). Bauchspeicheldrüse. Inselapparat ausgenommen. Spezielle pathologische Anatomie. Berlin.

[2] Stolte M, Weiss W (1982). A special form of segmental pancreatitis: groove pancreatitis. Hepatogastroenterology.

[3] Becker V, Mischke U (1991). Groove pancreatitis. Int J Pancreatol.

[4] Adsay NV, Zamboni G Paraduodenal pancreatitis: a clinico-pathologically distinct entity unifying “cystic dystrophy of heterotopic pancreas”, “para-duodenal wall cyst”, and “groove pancreatitis”. Semin Diagn Pathol.

[5] Zamboni G, Capelli P, Scarpa A, Bogina G, Pesci A, Brunello E, Klöppel G (2009). Nonneoplastic mimickers of pancreatic neoplasms. Arch Pathol Lab Med.

[6] Manzelli A, Petrou A, Lazzaro A (2011). Groove pancreatitis: a mini-series report and review of the literature. JOP.

[7] Shudo R, Yazaki Y, Sakurai S (2002). Groove pancreatitis: report of a case and review of the clinical and radiologic features of groove pancreatitis reported in Japan. Intern Med.

[8] Yamaguchi K, Tanaka M (1992). Groove pancreatitis masquerading as pancreatic carcinoma. Am J.

[9] Ray S, Ghatak S, Misra D (2017). Groove pancreatitis: report of three cases with brief review of literature. Indian J Surg.

[10] Blasbalg R, Baroni RH, Costa DN, Machado MC (2007). MRI features of groove pancreatitis. AJR Am J Roentgenol.

[11] Iglesias García J, Lariño Noia J, Domínguez Muñoz JE (2009). Endoscopic ultrasound in the diagnosis and staging of pancreatic cancer. Rev Esp Enferm Dig.

[12] Gabata T, Kadoya M, Terayama N, Sanada J, Kobayashi S, Matsui O (2003). Groove pancreatic carcinomas: radiological and pathological findings. Eur Radiol.

[13] Chantarojanasiri T, Isayama H, Nakai Y (2017). Groove pancreatitis: endoscopic treatment via the minor papilla and duct of Santorini morphology. Gut Liver.

[14] Isayama H, Kawabe T, Komatsu Y (2005). Successful treatment for groove pancreatitis by endoscopic drainage via the minor papilla. Gastrointest Endosc.

[15] Casetti L, Bassi C, Salvia R (2009). "Paraduodenal" pancreatitis: results of surgery on 58 consecutives patients from a single institution. World J Surg.

